# Lung Cancer Cells That Survive Ionizing Radiation Show Increased Integrin α2β1- and EGFR-Dependent Invasiveness

**DOI:** 10.1371/journal.pone.0070905

**Published:** 2013-08-08

**Authors:** Xue Li, Seiichiro Ishihara, Motoaki Yasuda, Takeshi Nishioka, Takeomi Mizutani, Masayori Ishikawa, Kazushige Kawabata, Hiroki Shirato, Hisashi Haga

**Affiliations:** 1 Transdisciplinary Life Science Course, Faculty of Advanced Life Science, Hokkaido University, Sapporo, Japan; 2 Department of Oral Pathobiological Science, Graduate School of Dental Medicine, Hokkaido University, Sapporo, Japan; 3 Department of Biomedical Sciences and Engineering, Faculty of Health Sciences, Hokkaido University, Sapporo, Japan; 4 Department of Medical Physics, Graduate School of Medicine, Hokkaido University, Sapporo, Japan; 5 Department of Radiology, Graduate School of Medicine, Hokkaido University, Sapporo, Japan; University of Pecs Medical School, Hungary

## Abstract

Ionizing radiation (IR)-enhanced tumor invasiveness is emerging as a contributor to the limited benefit of radiotherapy; however, its mechanism is still unclear. We previously showed that subcloned lung adenocarcinoma A549 cells (P cells), which survived 10 Gy IR (IR cells), acquired high invasiveness *in vitro*. Here, we tried to identify the mechanism by which IR cells increase their invasiveness by examining altered gene expression and signaling pathways in IR cells compared with those in P cells. To simulate the microenvironment *in vivo*, cells were embedded in a three-dimensional (3D) collagen type I gel, in which the IR cells were elongated, while the P cells were spherical. The integrin expression pattern was surveyed, and expression levels of the integrin α2 and β1 subunits were significantly elevated in IR cells. Knockdown of α2 expression or functional blockade of integrin α2β1 resulted in a round morphology of IR cells, and abrogated their invasion in the collagen matrix, suggesting the molecule’s essential role in cell spread and invasion in 3D collagen. Epidermal growth factor receptor (EGFR) also presented enhanced expression and activation in IR cells. Treatment with EGFR tyrosine kinase inhibitor, PD168393, decreased the ratio of elongated cells and cell invasiveness. Signaling molecules, including extracellular signal-regulated kinase-1/2 (Erk1/2) and Akt, exhibited higher activation in IR cells. Inhibition of Akt activation by treating with phosphoinositide 3-kinase (PI3K) inhibitor LY294002 decreased IR cell invasion, whereas inhibition of Erk1/2 activation by mitogen-activated protein kinase kinase (MEK) inhibitor U0126 did not. Our results show that integrin α2β1 and EGFR cooperatively promote higher invasiveness of IR-survived lung cancer cells, mediated in part by the PI3K/Akt signaling pathway, and might serve as alternative targets in combination with radiotherapy.

## Introduction

Lung cancer is the leading cause of cancer-related mortality throughout the world, with non-small cell lung cancer (NSCLC) accounting for the majority of cases. Treatment options for NSCLC include surgery, chemotherapy, radiotherapy, and sequential or concurrent combination therapy [1]. Radiotherapy is the medical use of ionizing radiation (IR), and is considered a non-invasive local treatment, affecting mainly the cells and tissues that are situated inside the beam of IR. Without a doubt, it has been proven as a fundamental tool available in the battle against cancer.

However, increasing experimental data suggest that, under circumstances not yet understood, radiotherapy of the primary tumor might favor metastasis, which may explain why better local control of radiation fails to translate into longer survival time, free of distant metastases [2]. Therefore, in addition to considerable efforts in enhancing radiosensitivity [3]–[6], the identification of molecules and the mechanisms of IR-induced metastatic cancer progression are required for improving the efficacy of radiotherapy and patient survival rate. Many studies have demonstrated that irradiation can promote invasion and/or metastasis by upregulating the expression of genes and activation of signaling pathways that are involved in the metastatic process. Among them, cell surface receptors, such as integrins and growth factor receptors, are often altered by IR and are capable of activating many different signaling pathways with multiple cellular responses. For instance, expression levels of integrin αvβ3 in glioma cells [7] and α5β1 in pancreatic cancer [8] are upregulated by IR, facilitating both cell migration and invasion. Integrin α3β1 is overexpressed after IR, promoting the migration of meningioma cells via focal adhesion kinase (FAK) and extracellular signal-regulated kinase (ERK) [9]. Our group [10] and others [11] showed a pivotal role of integrin β1 in IR-induced invasiveness in lung cancer and medulloblastoma, respectively. IR can also enhance invasion through activation of the epidermal growth factor receptor (EGFR) and insulin-like growth factor receptor 1 (IGFR1) [12], [13], and secretion of the hepatocyte growth factor (HGF) [14].

Herein, we sought to better understand the mechanism underlying the increased invasiveness of lung cancer cells that survived IR. We demonstrated that integrin α2β1 is selectively upregulated in IR cells, and is required for the aggressive phenotype and invasion of IR cells in the three-dimensional (3D) collagen gel. EGFR was also overexpressed and more active in IR cells, contributing to IR invasiveness as well. Investigation of several important signaling molecules showed activation of extracellular signal-regulated kinase-1/2 (Erk1/2) and Akt in IR cells, but only phosphoinositide 3-kinase (PI3K)/Akt mediated the invasive signaling transduction from integrin α2β1 and EGFR. Understanding how IR promotes the invasion of cancer cells may provide insight into metastasis and potential therapeutic targets to prevent the recurrence of secondary tumors after radiotherapy.

## Materials and Methods

### Cell Culture

Human lung adenocarcinoma cell line A549 was obtained from the American Type Culture Collection (ATCC; Manassas, VA). P cells and IR cells were generated as previously published [10]. Both cell lines were maintained in Dulbecco’s Modified Eagle’s Medium (DMEM; Sigma, St. Louis, MO) supplemented with 10% fetal bovine serum (FBS; Equitech-Bio, Kerrville, TX) and 1% antibiotic mixture of penicillin/streptomycin (Sigma). Cells were maintained in a humidified incubator at 37°C with 5% CO_2_.

### Reagents

EGFR kinase inhibitor PD168393 (Calbiochem, Merck KGaA, Damstadt), PI3K inhibitor LY294002 (Sigma-Aldrich, St. Louis, MO), and mitogen-activated protein kinase kinase (MEK) inhibitor U0126 (Cell Signaling Technology, Beverly, MA) were used at the indicated concentration in DMSO. A function-blocking antibody against integrin α2β1 (BHA2.1) was purchased from Millipore (Billerica, MA). Western blotting antibodies specific for the integrin α2 and β1 subunits were purchased from BD BioScience (San Jose, CA). The p-EGFR antibody (Tyr1068) was purchased from Signalway Antibody (College Park, Maryland). Antibodies specific to EGFR, Akt, p-Akt (Ser473), p44/42 Raf-mitogen-activated protein kinase MAPK (Erk1/2), p-p44/42 MAPK (Erk1/2) (Thr202/Tyr204), signal transducer and activator of transcription 3 (Stat3), p-Stat3 (Ser727), p38 MAPK, and p-p38 MAPK (Thr180/Tyr182) were purchased from Cell Signaling Technology (Beverly, MA). GAPDH antibody was purchased from Ambion (Austin, TX). MFP488 phalloidin was purchased from Mo Bi Tec (Molecular Biologische Technologie, Göttingen).

### 3D Collagen Culture

A 1.6 mg/mL collagen solution was prepared by mixing 3 mg/mL pig collagen type I-P solution (Nitta Gelatin, Osaka), 2.6× DMEM medium (Sigma), and buffer (Nitta Gelatin) at a ratio of 7∶5:1 on ice. A 30-mm dish was first coated with 150 µL of collagen solution and allowed to polymerize at 37°C for 30 min, then rinsed with medium. Then, 10 µL of 2×10^5^ cells in suspension was mixed thoroughly with 150 µL of collagen solution and plated on the lower layer of collagen gel. After collagen polymerization at 37°C for 30 min, the cell-collagen mixture was covered with 2 mL of FBS-containing medium and cultured at 37°C and 5% CO_2_ for further analysis. For morphology analysis and time-lapse observation, a glass dish was substituted for the plastic dish. For easier observation of cell movement in the same plane, gel-sand culture was used. Cells were first plated and allowed to adhere onto the lower gel and, after 16 h, the upper gel was overlaid and polymerized at 37°C for 30 min. Cells were maintained in 2 mL of FBS-containing medium at 37°C and 5% CO_2_.

### Cell Morphology Analysis

Cell morphology was analyzed after being in the 3D collagen gel for 24 h. When indicated, inhibitors or antibodies were added to the medium. Phase contrast images were taken randomly from 4 fields per sample, and the percentage of elongated cells was determined from at least 3 independent experiments including over 100 individual cells. A cell was considered elongated when its longest dimension was twice the shortest dimension, and when it showed at least one protrusion, as previously reported [15].

### Time-lapse Microscopy and Quantification of the Speed of Cell Invasion

2×10^4^ cells were cultured by 3D gel-sand assay for 24 h, and observed in a chamber at 37°C by a phase-contrast microscope (TE300, Nikon Instech, Tokyo). Images of randomly chosen cells were taken every 5 min for 6 h. For inhibition experiments, inhibitors or antibodies were added into the culture medium after gel-overlay when indicated. To quantify the speed of cells, we tracked the movements of individual cells by Image-Pro software (Media Cybernetics Inc., Silver Spring, MD). The cell invasion speed was calculated as distance (µm) per minute from at least 3 independent experiments including 50 individual cells.

### 3D Spheroid Invasion Assay

Spheroids were produced using the Gravity Plus system (InSphero, Zurich, Switzerland) according to the manufacturer’s instructions. Briefly, 40 µL of cell suspension containing 10^3^ cells was seeded into each well of the plate for 4 d, and spheroids were transferred onto collagen gel and overlaid soon thereafter. After being on gelatin at 37°C for 30 min, medium with FBS was added, and cells were cultured for 24 h. When indicated, inhibitors or antibodies were added during culture. Then, cells were fixed with 4% paraformaldehyde in PBS, permeabilized with 0.5% Triton X-100 in PBS, and stained with MFP488 phalloidin. Fluorescence images were obtained by confocal laser scanning microscopy (C1 confocal imaging system; Nikon Instech., Tokyo). The perimeter and the area of spheroids were determined by ImageJ software (National Institutes of Health, Bethesda, Maryland) as previously reported [16]. In brief, change the image to 8-bit type, and use the threshold function to convert areas of interest to saturated black areas in a uniform manner to have a binary (black&white) image. Then exclude all particles less than 3 pixels in size and remove any artifacts by comparing the binary image to the fluorescence pictures. Use the set measurements dialog box to specify area and perimeter. Use the analyze particle dialog box to measure all particles and to generate a “particle report” for each image in which the area and perimeter of individual particles and the area of the sum of individual particles is documented. The aspect ratio was calculated from perimeter^2^/[4π(area)]. A higher aspect ratio means a more irregular, infiltrating spheroid structure. Results were determined from 3 independent experiments carried out in triplicate.

### Western Blotting

Cells in 3D collagen culture were fixed in 500 µL ice-cold Trichloroacetic acid (TCA) for 3 min, and digested with 200 µL 0.1% collagenase at 37°C for 1 h. Cell pellets were collected by centrifugation at 14000 rpm for 2 min, resuspended in 100 µL Laemmli buffer, and heated at 95°C for 5 min, prior to lysates being sonicated for 30 sec and stored at −20°C until use. To perform western blotting, cell lysates were separated on a 12% SDS-polyacrylamide gel, and transferred to a polyvinylidene difluoride membrane (Millipore, Bedford, MA). The membrane was blocked with 5% reconstituted skim milk powder in TBST solution (10 mM Tris–HCl containing 150 mM NaCl and 0.05% Tween 20, pH 7.5). The blots were incubated with primary antibodies diluted in TBST or Can Get Signal Immunoreaction Enhancer Solution 1 (Toyobo, Osaka) at 4°C overnight. After washing with TBST, horseradish peroxidase-conjugated secondary antibodies diluted in TBST or Can Get Signal Immunoreaction Enhancer Solution 2 were applied and the blots were developed by the Enhanced Chemiluminescence Detection System (Perkin Elmer, Waltham, MA). Levels of GAPDH immunocomplexes were used as an internal standard for equal loading. Quantification of signal intensity was performed using the ImageJ software and normalized to the control value.

### RT-PCR

Cells were lysed with Tripure (Roche Applied Science, Indianapolis, IN) for RNA extraction, and the reverse transcription reaction was performed by ReverTra Ace qPCR RT Kit (Toyobo). For cells cultured in 3D collagen gel, extraction was performed twice. PCR was performed with Taq Polymerase in ThermoPol Buffer (NEB, Ipswich, MA). Primers were as follows: integrin α1∶5′-GCCTCCTTTCTTGCTGTGTC-3′ (Forward), 5′-TGGGTGCTTATTGGTTCTCC-3′ (Reverse); integrin α2∶5′-GAGCACCAGCAACAAAGTGA-3′ (Forward), 5′-CGGGTGTGTGTTCTGACATC-3′ (Reverse); integrin α4∶5′-GAGATTTTCCCCTTGCATGA-3′ (Forward), 5′-GAGTGCAATGCAGACCTTGA-3′ (Reverse); integrin α5∶5′-CACAGAGTTGCCCCGAGCACA-3′ (Forward), 5′-GCAGGGCTAGTGCCAGGGTTT-3′ (Reverse); integrin β1∶5′-AATGAAGGGCGTGTTGGTAG-3′ (Forward), 5′-CCTCGTTGTTCCCATTCACT-3′ (Reverse); and GAPDH: 5′-ACCACAGTCCATGCCATCAC-3′ (Forward), 5′-TCCACCACCCTGTTGCTGTA-3′ (Reverse).

### Quantitative Real-time PCR (qRT-PCR)

qRT-PCR was performed by PikoReal (Thermo Scientific, Waltham, MA) according to the manufacturer’s instructions. Briefly, total RNA (1 µg) was reverse transcribed using the specific primers as follows: integrin α2∶5′-CACAGAGTTGCCCCGAGCACA-3′ (Forward), 5′-GCAGGGCTAGTGCCAGGGTTT-3′ (Reverse); integrin β1∶5′-GACGCCGCGCGGAAAAGATG-3′ (Forward), 5′-GCACCACCCACAATTTGGCCC-3′ (Reverse); EGFR: 5′-CGCAGATAGTCGCCCAAAG-3′ (Forward), 5′-CCATCAGGGCACGGTAGAA-3′ (Reverse); and β-actin: 5′-GAGCCTCGCCTTTGCCGATCC-3′ (Forward), 5′-ACATGCCGGAGCCGTTGTCG-3′ (Reverse), which was used as a reference gene for normalization.

### Small Interfering RNA (siRNA) Transfection

Cells were transfected with siRNA against the integrin α2 target sequence 5′-AACCAAAGAAGAAATGATTGTAG-3′ (sense sequence, siα2-1) or 5′-AACAAGAATGCTCAGATAATTCT-3′ (sense sequence, siα2-2) using Lipofectamine RNAiMAX Reagent (Invitrogen, Carlsbad, CA). A siRNA against the Azami Green target sequence 5′-AGCAGATATTCAGGACTATTTCA-3′ (sense sequence) was used as a negative control.

### Proliferation Assay

2×10^4^ cells were cultured in 3D collagen gel in 24-well plate, and treated with inhibitors or antibodies when indicated during the culture. Medium with or without inhibitors or antibodies were changed every two days. The cells in 3D collagen culture were fixed in 200 µL ice-cold TCA for 3 min, and digested with 200 µL 0.1% collagenase at 37°C for 1 h, pipetted thoroughly and continue to be digested for another 1 h. Cell pellets were collected by centrifugation, and resuspended with PBS. Cell density was determined with a hemocytometer. All determinations were performed in triplicate in 3 independent experiments.

### Statistical Analysis

Each experimental condition was repeated at least 3 times. The data are expressed as mean ± S.D. Statistical analysis was performed using the Student’s *t*-test, and a P value ≤0.05 was considered significant.

## Results

### IR Cells Present Higher Invasive Ability

To examine whether IR can promote cancer cell invasion, cell phenotype was first compared between P and IR cells. Unlike similar morphology on 2D stiff substrate, cell morphologies differ significantly when embedded in a 3D collagen gel, where P cells are spherical; IR cells are more elongated with protrusions [10].

**Figure 1:**
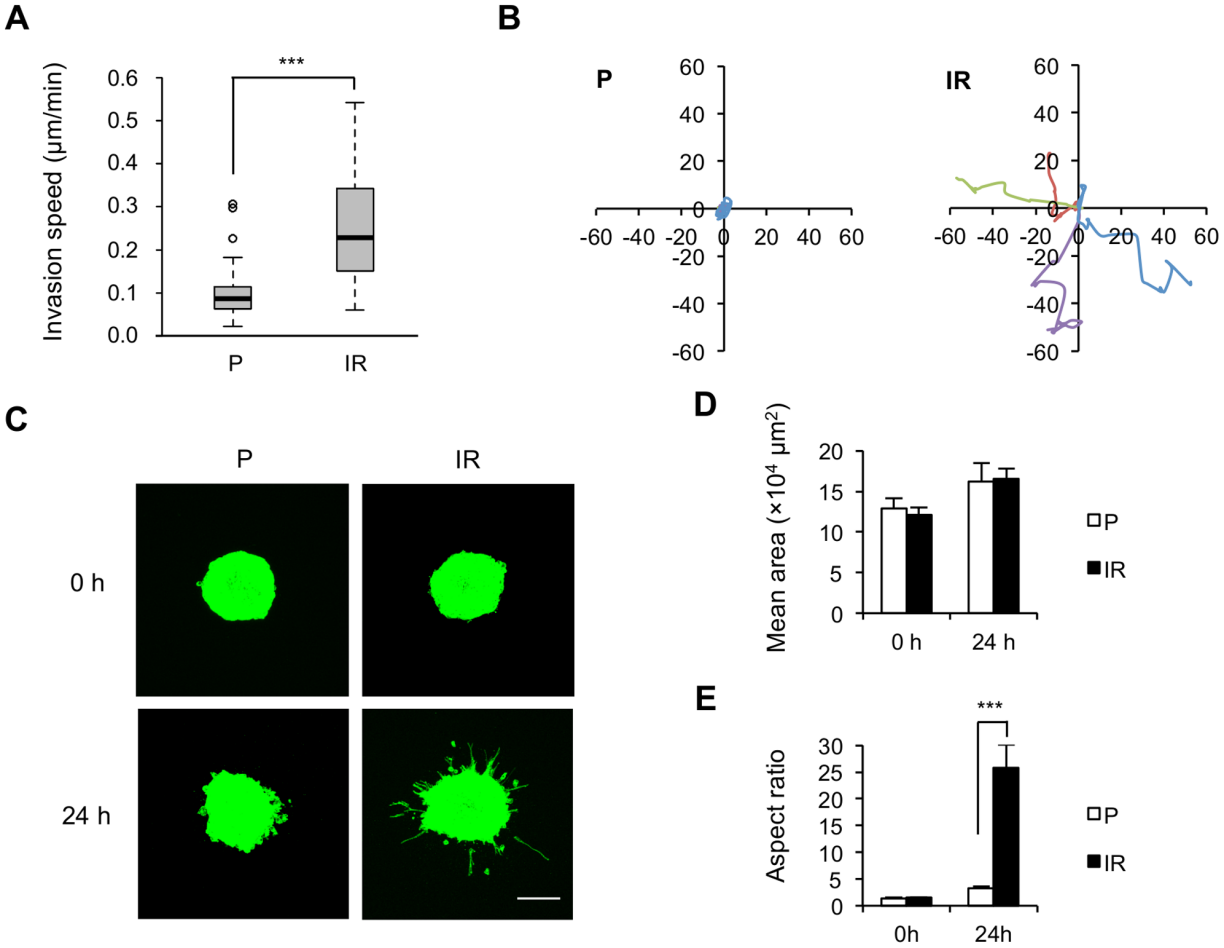
IR cells present increased invasive ability in a 3D collagen gel. (A) Quantification of invasion speed in P and IR cells presented as mean values ± S.D, ***p<0.001. (B) Diagrams representing the invasion trajectories of 4 representative cells from P and IR cells in 3D collagen gel-sand covered for 6 h. Cell origins were set as (0,0), and the scale unit is µm. (C) Confocal images of representative MFP488 phalloidin-stained P and IR spheroids in collagen gel at 0 h or 24 h. Scale bar, 200 µm. (D) Quantification of the area of spheroids by ImageJ software. (E) The aspect ratio of spheroids was calculated from perimeter^2^/[4π(area)]. Results are presented as mean values ± S.D (***p<0.001) from 3 independent experiments in triplicate.

Quantification of invasion speed of individual cells showed that IR cells moved faster by about two-fold than P cells in collagen gel (Fig. 1A). Moreover, trajectories of IR cells were longer and more directed than those of P cells, with cells often turning around (Fig. 1B). Increased invasiveness of IR cells was further confirmed by 3D spheroid invasion assay to mimic the characteristic of tumors *in vivo* (Fig. 1C). The results show that, after embedded in collagen gel for 24 h, both P and IR spheroids increased in volume by about 20–40% (Fig. 1D), whereas IR spheroids extended massive protrusions, with some cells having already escaped from the body, and presented as a higher aspect ratio than that of P cells (Fig. 1E), suggesting a higher invasiveness of IR cells in microtissues.

### Integrin α2β1 is Overexpressed in IR Cells, and is Required for the Elongation and Invasiveness of IR Cells in 3D Collagen

**Figure 2:**
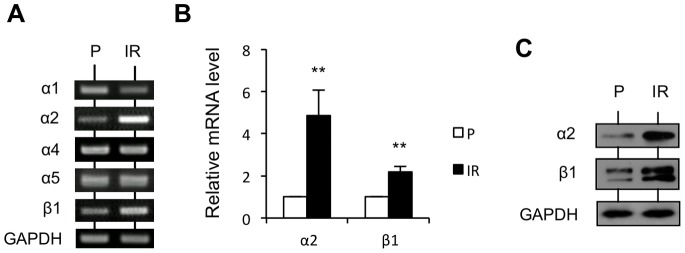
Integrin α2 and β1 subunits are specifically upregulated in IR cells. (A) Semi-quantitative analysis of the mRNA levels of integrin subunits, including α1, α2, α4, α5, and β1, from P and IR cells by RT-PCR. (B) Quantitative analysis of the mRNA levels of integrin α2 and β1 subunits from P and IR cells by qRT-PCR. Intensity of signals was quantified by densitometry and normalized with β-actin. Representation is mean value ± S.D of relative mRNA level (**p<0.01) from 3 independent experiments, indicated as fold change relative to P cells. (C) Analysis of the protein levels of integrin α2 and β1 subunits from P and IR cells cultured in 3D collagen gel by western blotting. GAPDH was used as a loading control.

**Figure 3:**
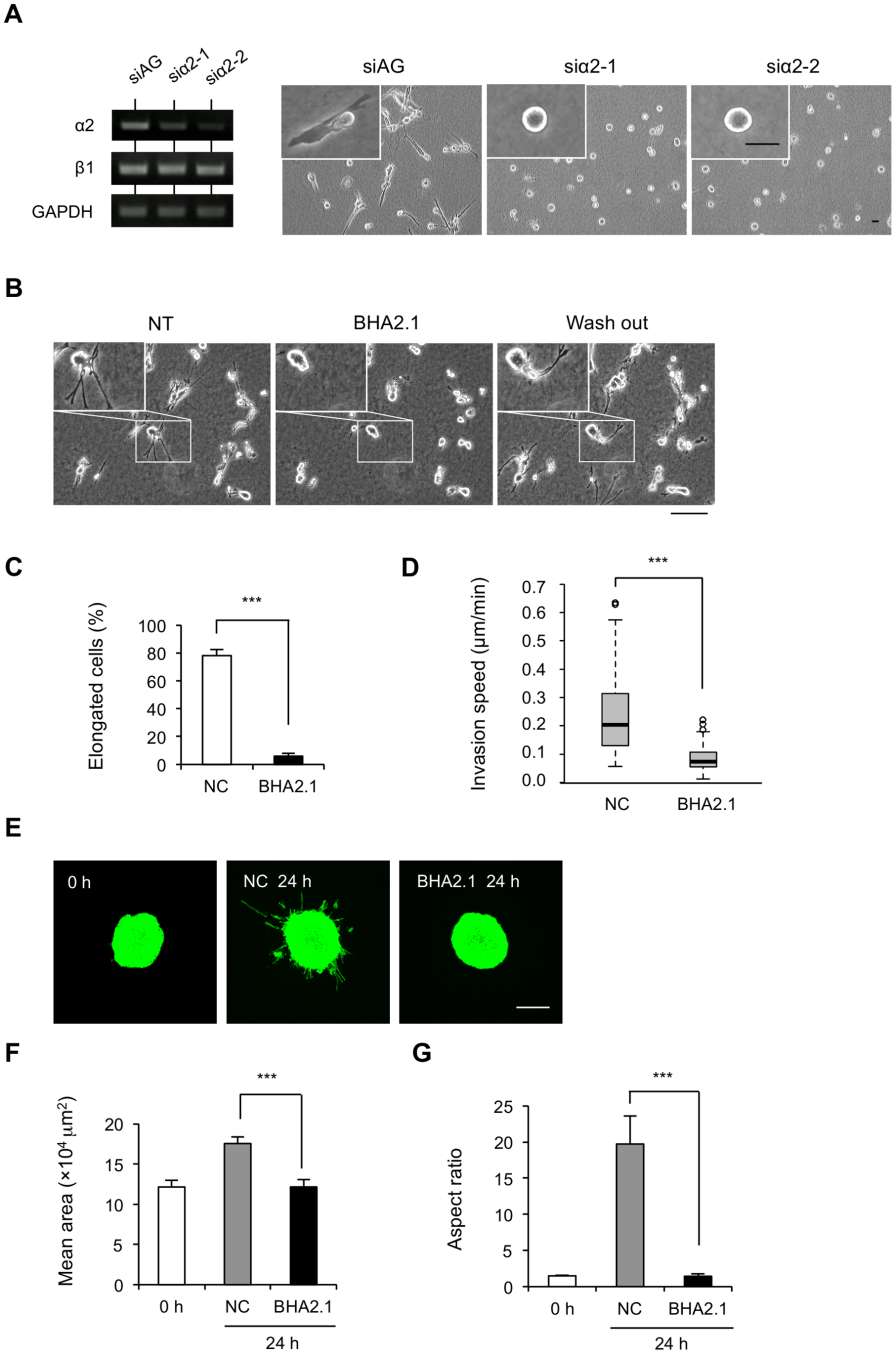
Integrin α2β1 is required for IR cell invasion. (A) Knockdown of the integrin α2 subunit in IR cells resulted in a round morphology in 3D collagen gel. IR cells that were transfected with 2 siRNAs specific to integrin α2 (siα2-1 or siα2-2), or a specific siRNA targeted to Azami-Green (siAG) as a control, were transferred to a 3D collagen gel and cultured for 24 h. Left: RT-PCR results to verify the effect of specific knockdown of integrin α2. Right: representative images of cell morphologies. Magnification of individual cell represented by the white boxes. Scale bar, 20 µm. (B) Functional blocking of integrin α2β1 induced a reversible retraction of protrusions and invasiveness of IR cells. Time-lapse observation of IR cells treated with BHA2.1 in 3D collagen gel-sand. The representative images of IR cells that were non-treated (NT), treated with BHA2.1 (BHA2.1), or washed out (Wash out) with fresh medium are shown. White boxes outline the certain area that magnified. Scale bar, 100 µm. (C) Cell morphology analysis of BHA2.1-treated IR cells versus controls in 3D collagen gel by quantifying the percentage of elongated cells in total, expressed as mean values ± S.D (***p<0.001) from 3 independent experiments including about 100 cells. (D) Quantification of speed in P and IR cells in 3D collagen gel-sand for 6 h, expressed as mean values ± S.D (***p<0.001) from 3 independent experiments including about 50 cells. (E) Confocal images of representative of MFP488 phalloidin-stained P and IR spheroids in collagen gel at 0 h or 24 h. Scale bar, 200 µm. (F) Quantification of the area of spheroids by ImageJ software. (G) The aspect ratio of spheroids was calculated from perimeter^2^/[4π(area)]. Results are presented as mean values ± S.D (***p<0.001) from 3 independent experiments in triplicate.

Integrins are cell surface-adhesive receptors formed by α and β subunits, which bind to extracellular matrix (ECM) proteins. Integrin-mediated adhesion to the ECM triggers intracellular signaling pathways to modulate cell morphology, migration, invasion, proliferation, and survival [17]. The dramatic morphological change of IR cells compared to P cells when surrounded by a collagen matrix encouraged us to investigate the integrin expression pattern. In our previous study, we showed that knockdown of integrin β1 by siRNA or treatment with its inhibitory antibody AIIB2 induced spherical morphology of IR cells in 3D collagen gel, similar to P cells [10]. Given that collagen type I and fibronectin (sequestered from the FBS in the medium and secreted from the cells) are the main ECM components in our collagen gel model, the expression pattern of integrins, including α1β1, α2β1, α4β1, and α5β1, was investigated by RT-PCR. Among them, α1β1 and α2β1 are reported as the main collagen receptors, whereas α4β1 and α5β1 are reported as the main fibronectin receptors [18]. The results of RT-PCR indicate that, in IR cells, the transcription levels of α2 and β1 increased, the level of α1 decreased, and there was no obvious change in the levels of α4 and α5 (Fig. 2A). The results of qRT-PCR further confirmed that the transcription level of α2 was enhanced by 4.8-fold, and that of β1 was enhanced by 2.2-fold (Fig. 2B). In addition, western blotting was carried out to detect their protein levels, and a similar elevation was observed (Fig. 2C). These results suggest that integrin α2β1 might play an important role in the altered interaction between IR cells and the ECM. To confirm whether the elevated expression of integrin α2β1 is essential for IR cell invasiveness, knockdown of α2 expression in IR cells by two kinds of siRNA specific to integrin α2 was carried out, and the effect was verified by RT-PCR (Fig. 3A, left). Indeed, knockdown of α2 impaired IR cell elongation (Fig. 3A, right) and invasion in collagen gel (Movie S1, S2).

Since integrins directly bind components of the ECM and provide the traction necessary for cell motility and invasion, we considered whether the interaction between integrin α2β1 and the ECM was important for IR cell invasion. The function-blocking antibody BHA2.1 (400 ng/mL) that recognizes the I domain of α2, the binding site for collagens, was used to treat IR cells in the gel. Time-lapse observation showed that blocking the activation of integrin α2β1 induced both the contraction of cell protrusions and low invasiveness soon after treatment, and removing the antibody by the addition of fresh medium restored invasion (Fig. 3B, Movie S3). BHA2.1 treatment significantly decreased the ratio of elongated phenotype (Fig. 3C) and invasion speed in IR cells (Fig. 3D), and abolished spheroid invasion (Fig. 3E–3G), which suggests that functional integrin α2β1 is required for IR cell invasion.

### Increased EGFR Expression and Activation in IR Cells is Involved in IR Cell Invasion

**Figure 4:**
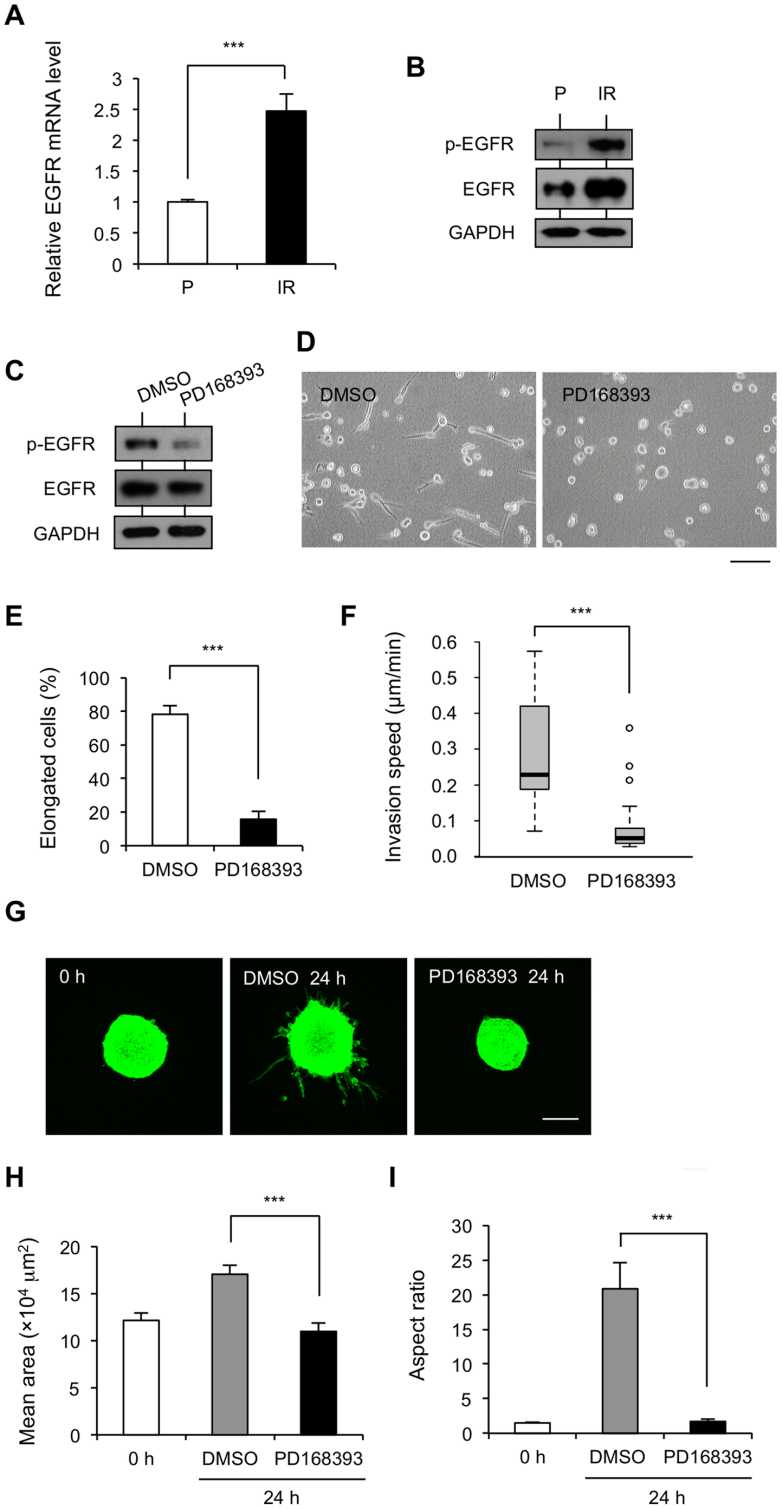
EGFR is overexpressed and activated in IR cells to promote IR cell invasion. (A) Quantitative analysis of EGFR mRNA levels from P and IR cells by qRT-PCR, represented as mean values ± S.D of relative mRNA level (***p<0.001) from 3 independent experiments, indicated as fold change relative to P cells. (B) Analysis of protein levels of total EGFR and phosphorylated EGFR (Tyr1068) by western blotting. Intensity of signals were quantified by densitometry and normalized with GAPDH. Results are represented as mean values ± S.D of relative protein level (**p<0.01) from 3 independent experiments, indicated as fold change relative to P cells. (C–E) Inhibition of EGFR activation induced a round morphology of IR cells. IR cells in 3D collagen gel were treated with 10 µM EGFR inhibitor PD168393 or DMSO as a control for 24 h. (C) Expression analysis of phosphorylated EGFR (Tyr1068) and total EGFR from PD168393- and DMSO-treated samples by western blotting. GAPDH was used as a control. (D) Representative images of cell morphologies of PD168393-treated samples versus the DMSO-treated control. (E) The percentage of elongated cells was quantified from 3 independent experiments including about 100 cells, ***p<0.001. (F) Quantification of speed in DMSO- or PD168393-treated IR cells in 3D collagen gel-sand for 6 h are represented as mean values ± S.D (***p<0.001) from 3 independent experiments including about 50 cells. (G) Confocal images of representative MFP488 phalloidin-stained IR spheroids in collagen gel for 0 h and IR spheroids treated with DMSO or PD168393 for 24 h. Scale bar, 200 µm. (H) Quantification of the area of spheroids by ImageJ software. (I) The aspect ratio of spheroids was calculated from perimeter^2^/[4π(area)]. Results are represented as mean values ± S.D (***p<0.001) from 3 independent experiments in triplicate.

EGFR is a receptor tyrosine kinase that is frequently overexpressed or harbors constitutively active mutations in NSCLC [19]. Thus, we checked whether any alterations of EGFR occurred in IR cells. Surprisingly, both EGFR transcriptional level and protein level were much elevated in IR cells, compared with those in P cells (Fig. 4A, 4B). A consistently high level of EGFR activation on the signaling-related residue Tyr1068 was also observed in IR cells without any stimulation by EGFR ligand (Fig. 4B). Therefore, a specific inhibitor targeting the tyrosine kinase of EGFR, PD168393 (10 µM), was used to treat IR cells, and was shown to decrease the phosphorylation of EGFR (Fig. 4C), the ratio of elongated IR cells (Fig. 4D, 4E), and the invasion speed (Fig. 4F). Like integrin α2β1 inhibition, PD168393-treated IR spheroids remained regular spheroids without volume expansion (Fig. 4G, 4H) or protrusion (Fig. 4G, 4I). These results support the hypothesis that the EGFR signaling pathway is involved in the increased invasiveness of IR cells.

### Integrin α2β1 and EGFR Promote IR Cell Invasion Partially through PI3K/Akt

**Figure 5:**
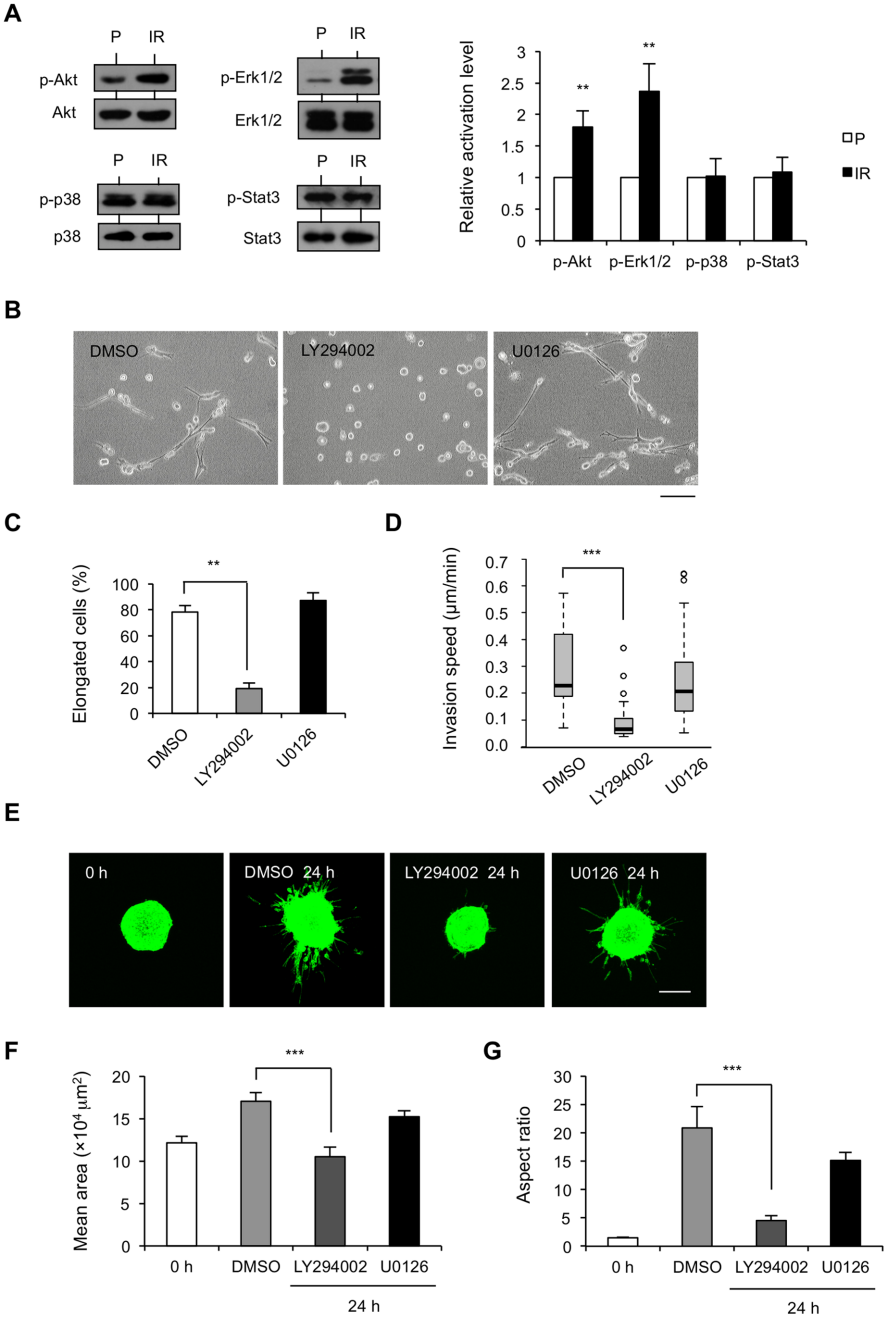
PI3K/Akt but not MEK/Erk1/2 mediates IR cell invasion. (A) Expression analysis of the total and phosphorylated forms of Akt (Ser473), Erk1/2 (Thr202/Tyr204), p38 (Thr180/Tyr182) and Stat3 (Ser727) by western blotting. Intensity of signals was quantified by densitometry and normalized with GAPDH. Results are represented as mean values ± S.D of relative protein level (**p<0.01) from 3 independent experiments, indicated as fold change relative to P cells. (B–C) Effects of inhibition of Akt and Erk1/2 activation on morphology of IR cells. (B) Phase contrast images of PI3K inhibitor LY294002 (50 µM) or MEK inhibitor U0126 (10 µM) treated IR cells versus DMSO-treated IR cells for 24 h in 3D collagen gel (C) The percentage of elongated cells was quantified from 3 independent experiments including about 100 cells, ***p<0.001. (D) Quantification of speed in DMSO-, 50 µM LY294002-, or 10 µM U0126-treated IR cells in 3D collagen gel-sand for 6 h, represented as mean values ± S.D (***p<0.001) from 3 independent experiments including about 50 cells. (E) Confocal images of representative MFP488 phalloidin-stained IR spheroids in collagen gel for 0 h and IR spheroids treated with DMSO, LY294002, or U0126 for 24 h. Scale bar, 200 µm. (F) Quantification of the area of spheroids by ImageJ software. (G) The aspect ratio of spheroids was calculated from perimeter^2^/[4π(area)], and results are presented as mean values ± S.D (***p<0.001) from 3 independent experiments in triplicate.

**Figure 6:**
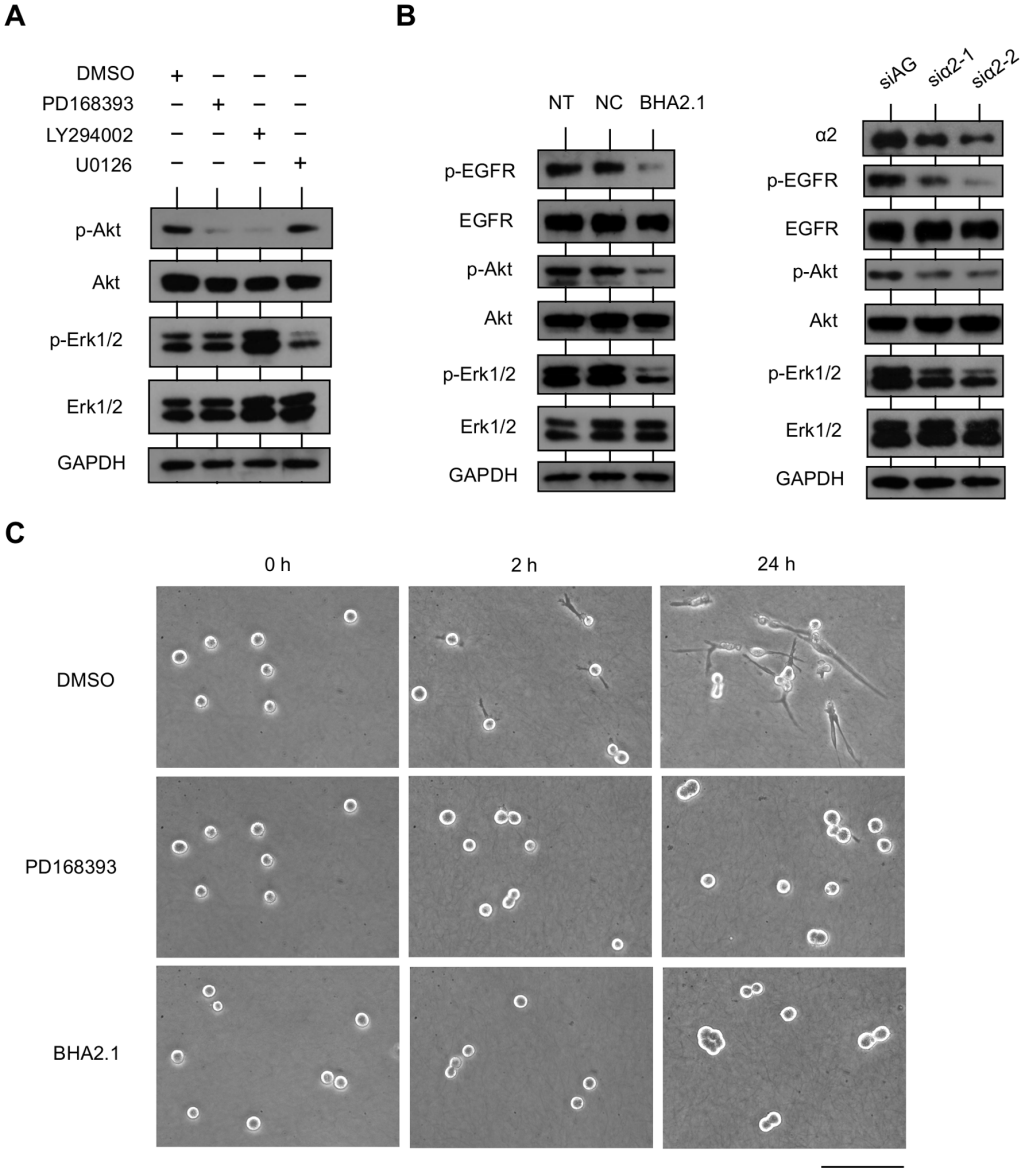
EGFR and integrin α2β1 coordinately regulate downstream signaling pathways responsible for IR cell invasion. (A) Regulation of EGFR in PI3K/Akt and MEK/Erk1/2 signaling pathways. IR cells in 3D collagen gel were treated with DMSO (control), EGFR inhibitor PD168393 (10 µM), PI3K inhibitor LY294002 (50 µM), or MEK inhibitor U0126 (10 µM) for 24 h. Cells were harvested, and the amounts of phosphorylated and total Akt (Ser473) and Erk1/2 (Tyr202/Tyr204) were analyzed by western blotting. (B) Regulation of integrin α2β1 on EGFR, PI3K/Akt, and MEK/Erk1/2 signaling pathways. Left: IR cells in 3D collagen gel were non-treated (NT), treated with non-function blocking antibody for integrin α2β1 (NC), or treated with function blocking antibody for integrin α2β1 (BHA2.1) for 24 h. Right: IR cells that transfected with siRNA specific to AzamiGreen (siAG) as a negative control or a siRNA specific to integrin α2 (siα2-1 or siα2-2) were transferred to 3D collagen gel and cultured for 24 h. Cells were harvested, and the amounts of integrin α2, phosphorylated and total EGFR (Tyr1068), Akt (Ser473), and Erk1/2 (Tyr202/Tyr204) were analyzed by western blotting. (C) Inhibition of EGFR or function blocking of integrin α2β1 in cells invading collagen. IR cell suspension in medium with DMSO, PD168393, or BHA2.1 was seeded on collagen gel. Phase contrast images for each sample at 0 h, 2 h, and 24 h are shown. DMSO-treated IR cells have already protruded into collagen at 2 h and present long protrusions at 24 h. In contrast, PD168393- or BHA2.1-treated IR cells simply adhere to the collagen with a spherical morphology.

To further identify the mechanism of the integrin α2β1- and EGFR-dependent IR cell invasion, we surveyed several important downstream signaling molecules that were regulated by integrin α2β1 and/or EGFR, including MEK/Erk1/2 [20], [21], PI3K/Akt [21], [22], Stat3 [23], and p38 MAPK [24], [25]. Among them, western blotting showed only Erk1/2 and Akt activation to be significantly upregulated in IR cells, with the formers’ total and phosphorylated protein levels on the residues necessary for signal transduction (Fig. 5A). To confirm whether their activation is related to IR cell invasiveness, specific inhibitors targeting their upstream kinases were used, including MEK inhibitor U0126 (10 µM) for Erk1/2 and PI3K inhibitor LY294002 (50 µM) for Akt. The activation of Akt and Erk1/2 was abrogated by decreased phosphorylation upon inhibition of their upstream molecules (Fig. 6A). Morphology analysis showed that LY294002 treatment decreased the percentage of elongated cells (Fig. 5C) and, thus, invasion speed (Fig. 5D), while U0126 treatment did not. Consistently, 3D spheroid invasion assay showed that IR cell invasion into collagen gel was suppressed only after treatment with LY294002, whereas U0126 had little effect (Fig. 5E, 5G), even though spheroid expansion was inhibited slightly (Fig. 5F). These results suggest the involvement of PI3K/Akt, but not MEK/Erk1/2, in invasive signal transduction in IR cells.

Since both PI3K/Akt and MEK/Erk1/2 signaling pathways could be activated by EGFR and integrin, we investigated which is responsible for their activation in IR cells. We found that Akt activation was downregulated by either inhibiting EGFR or blocking integrin α2 expression or α2β1 function (Fig. 6A, 6B). Although Erk1/2 is regarded as being regulated by EGFR [26], [27], decreased Erk1/2 activation was only observed upon specific integrin α2 silencing or functional blockade of integrin α2β1 (Fig. 6B).

The similar effect of integrin α2β1 and EGFR on Akt activation and IR cell invasiveness prompted us to study whether their overexpression and/or activation are dependent on each other. Knockdown of integrin α2 or functional blockade of integrin α2β1 suppressed activation of EGFR (Fig. 6B). On the other hand, inhibition of EGFR tyrosine kinase activity did not affect expression of α2 or β1 (data not shown), but attenuated cell protrusion into the collagen gel (Fig. 6C). These results suggest that expression and activation of integrin α2β1 are crucial for the activation of EGFR and downstream signaling, and EGFR activation might be necessary for integrin α2β1 function in mediating cell invasion into the collagen matrix, moreover, the switch to the invasive morphology of IR cells not only depends on the presence of collagen substrate for interaction with integrin α2β1 extracellular domain, but also depends on the intracellular signaling activation by integrin α2β1 cytoplasmic domain. Taken together, based on the results, it appears that integrin α2β1 and EGFR coordinately promote invasion of IR-survived cells, partially through the activation of PI3K/Akt signaling pathway.

## Discussion

Lung cancer is a common lethal cancer that is attributed with a high risk of metastatic dissemination. As a basic and important treatment for lung cancer, radiotherapy sometimes causes increased malignancy in the repopulated cancer cells. We initiated this study by aiming to identify the important molecules required for the increased invasiveness of IR-survived lung cancer cells to discover potential candidates that could be targeted in combination with radiotherapy. To decrease the possibility that cancer stem cells induce radioresistance [28], and for better analysis of IR-induced invasiveness, heterogeneous A549 cells were first screened as a relatively less invasive subclone to be parent cells. Then, P cells were subjected to a therapeutic dose of IR to mimic the clinical observation in which most of the cancer cells undergo apoptosis after IR exposure. The small fraction of cancer cells that survived was harvested as IR cells.

Invasive behavior was compared between P cells and IR cells in a fibrillar collagen matrix, the most abundant ECM component in the lung connective tissue [29], to mimic the *in vivo* environment. We found that P cells are spherical, whereas IR cells are elongated to favor their directional invasion in collagen. Quantification of individual cell movement and cell spheroid invasion in 3D collagen gel indicated higher invasiveness in IR cells compared to P cells, while the proliferation rates in the gel are similar (Fig. S1A). As our previous study [10] showed, integrin β1 is required for the increased invasive ability of IR cells. Screening of several integrin α subunits that ligate with β1 showed that the α2 subunit is specifically upregulated in IR cells. The overexpression and enhanced activity of integrin α2β1 were required for the long protrusion and invasion of IR cells. Recent work has underlined the implication of integrin α2β1 in cancer cell invasion and metastasis. For example, the expression of integrin α2β1 is upregulated in highly aggressive melanoma cells, mediating the reorganization of collagen I fibrils [30]. α2β1 integrin affects the metastatic potential of ovarian carcinoma spheroids by supporting disaggregation and proteolysis [31]. Reorganization of the integrin α2 subunit was suggested to control adhesion and invasion in prostate cancer [32]. It is worth noting that the integrin α2 subunit was identified as a human lung tumor-associated antigen, and its overexpression is considered directly involved in the pathogenesis of non-small cell tumors through its effects on invasion and/or metastasis [33]. In our study, increased expression of both the α2 and β1 subunits was observed in IR cells, suggesting a pivotal role of integrin α2β1 in the increased invasiveness after IR treatment. Interestingly, the mRNA level of the integrin α1 subunit decreases in IR cells. Several studies reported that integrin α1β1 and α2β1 might play contrasting roles in many aspects, such as collagen and collagenase gene expression [34], and EGFR activation [35], [36], which suggests that decreased expression of α1 integrin might also favor the increased invasiveness of IR cells.

In addition to integrin α2β1, a growth factor receptor that is often aberrant in NSCLC, EGFR, was found overexpressed and activated in IR cells. Although it has been demonstrated that advantages of EGFR inhibition on radiosensitization of cancer cells is mainly due to a reduction in cell proliferation and clonogenic survival [37], our results provided new evidence for the importance of EGFR inhibition. We showed here that EGFR expression and activation were elevated in lung cancer cells that survived IR, and this elevation was required for their increased invasiveness.

The roles of EGFR and integrin α2β1 in the activation of Akt were noted through its impaired activation after inhibition of EGFR or functional blockade of integrin α2β1. On the other hand, inhibition of PI3K/Akt resulted in similar spherical morphology and partially blocked the EGFR- and integrin α2β1-mediated invasion in IR cells. In contrast, the elongated phenotype and invasiveness of IR cells were not dependent on MEK/Erk1/2, even though Erk1/2 was also showed activation in IR cells. Alternatively, increased Erk1/2 activation in the presence of the PI3K inhibitor suggests the existence of a compensatory mechanism between PI3K/Akt and MEK/Erk1/2 signaling pathways, which has been implicated in other studies [38]–[40]. In addition, Erk1/2 activation was dependent on activation of integrin α2β1, but not EGFR, which is possibly related to the survival of IR cells upon the stress of IR, as other studies have suggested [41], [42]. However, direct inhibition of MEK/Erk1/2 may cause undesirable outcomes, such as augmenting EGFR-driven motility demonstrated in prostate cancer [27].

Recent work showed crosstalk between signaling pathways involving integrins and EGFR in cancer progression [43]–[45]. For example, physical association between integrin α2β1 and EGFR at cell-cell contact sites was reported in A431 cells with unknown biological function [36]. Expression of the integrin α2 subunit was selectively increased upon EGF-mediated EGFR activation in both A431 cells and A549 cells [46]. β1 integrin-silenced cells show defective activation of the EGFR signaling cascade, leading to decreased *in vitro* proliferation, enhanced sensitivity to cisplatin and gefitinib, impaired migration, and invasive behavior of A549 cells [45]. These observations support our hypothesis that integrin α2β1 and EGFR may coordinately regulate signal transduction responsible for IR cell invasion.

Finally, we also investigated whether the integrin α2β1/EGFR axis is also important for IR cell proliferation by performing proliferation assay with cells in 3D collagen gel (Fig. S1B, S1C). We found that IR cell proliferation was partially suppressed by integrin α2β1 and MEK/Erk1/2 inhibition, and totally blocked by EGFR and PI3K/Akt inhibition compared to the control after long time treatment. These results are consistent with other observations on the involvement of these molecules in cell proliferation, survival and anti-apoptosis [47]–[50]. However, under our experiment condition, cells were only treated with inhibitors or antibodies for 24 h to 30 h in/on 3D collagen gel, when cell proliferation was barely affected, whereas the cell morphology and invasive ability were affected substantially. And we found that during the first 24 h in collagen gel, cells start morphologic change and movement rather than proliferation.

EGFR is a promising target for combination with radiotherapy in many cancer types [51], [52]. Specific antibodies or small molecule inhibitors against EGFR have already been used for the treatment of NSCLC, and have improved progression-free and overall survival. However, despite initial response and long lasting remission, the development of secondary resistance inevitably leads to treatment failure [53].

In contrast to EGFR-targeting therapy, integrin inhibitors are not fully appreciated partially due to the lack of knowledge of the particular integrin that plays the dominant role in pathological microenvironments [54]. Integrin antagonists, including the αvβ3 and αvβ5 inhibitor cilengitide, have shown encouraging results in Phase II clinical trials, and cilengitide is currently being tested in a Phase III trial in patients with glioblastoma [55]. Our results point out that the integrin α2β1 is required for aggressive phenotype and increased invasiveness of repopulated lung cancer cells after irradiation, and its function blocking is sufficient to abrogate the IR cell invasion in 3D collagen matrix, supporting the rationale for combining integrin inhibitors with radiotherapy.

## Supporting Information

Figure S1 **Proliferation analysis.** (A) 2×10^4^ P and IR cells were cultured in 3D collagen gel in 24-well plate for 1 to 4 days to evaluate their proliferation rates. (B) 2×10^4^ IR cells were cultured in 3D collagen gel in 24-well plate for 1 to 4 days, treated with non-function blocking antibody for integrin α2β1 (NC), or treated with function blocking antibody for integrin α2β1 (BHA2.1). (C) 2×10^4^ IR cells were cultured in 3D collagen gel in 24-well plate for 1 to 4 days, treated with PD168393, LY294002 or U0126 versus DMSO control. Cell numbers are presented as mean values ± S.D (*p<0.05, **p<0.01, ***p<0.001) from 3 independent experiments performed in triplicate.
(TIF)

Movie S1 **Negative control for knockdown on IR cell invasion in 3D collagen gel-sand.** Time-lapse phase contrast observation of IR cells transfected with siRNA specifics to AzamiGreen (siAG) as a negative control cultured in a 3D collagen gel-sand for 12 h. Cells were transfected on a dish and, 24 h later, were transferred to gel-sand to allow cell spreading for 24 h, before being subjected to observation. Video time, 1 second = real time, 75 minutes; screen width, 650 µm.
(AVI)

Movie S2 **Knockdown of integrin α2 on IR cell invasion in 3D collagen gel-sand.** Time-lapse phase contrast observation of IR cells transfected with a siRNA specifics to integrin α2 (siα2-2) cultured in a 3D collagen gel-sand for 12 h. Cells were transfected on a dish and, 24 h later, were transferred to gel-sand to allow cell spreading for 24 h, before being subjected to observation. Video time, 1 second = real time, 75 minutes; screen width, 650 µm.
(AVI)

Movie S3 **The effect of integrin α2β1 functional blockade on IR cell invasion in 3D collagen gel-sand.** Time-lapse phase contrast observations of IR cells cultured in a 3D collagen gel-sand. IR cells were observed for 8 h (untreated condition). After observation, the cells were treated with BHA2.1 and observed for 6 h. After washing out the BHA2.1 with fresh medium, the cells were observed for 18 h. Video time, 1 second = real time, 75 minutes; screen width, 650 µm.
(AVI)
