# Supplementary figures and images for: Lung Cancer Cells That Survive Ionizing Radiation Show Increased Integrin α2β1- and EGFR-Dependent Invasiveness
Source: PLoS One. 2013 Aug 8;8(8):e70905. doi: 10.1371/journal.pone.0070905 (PMC3738636; doi:10.1371/journal.pone.0070905)

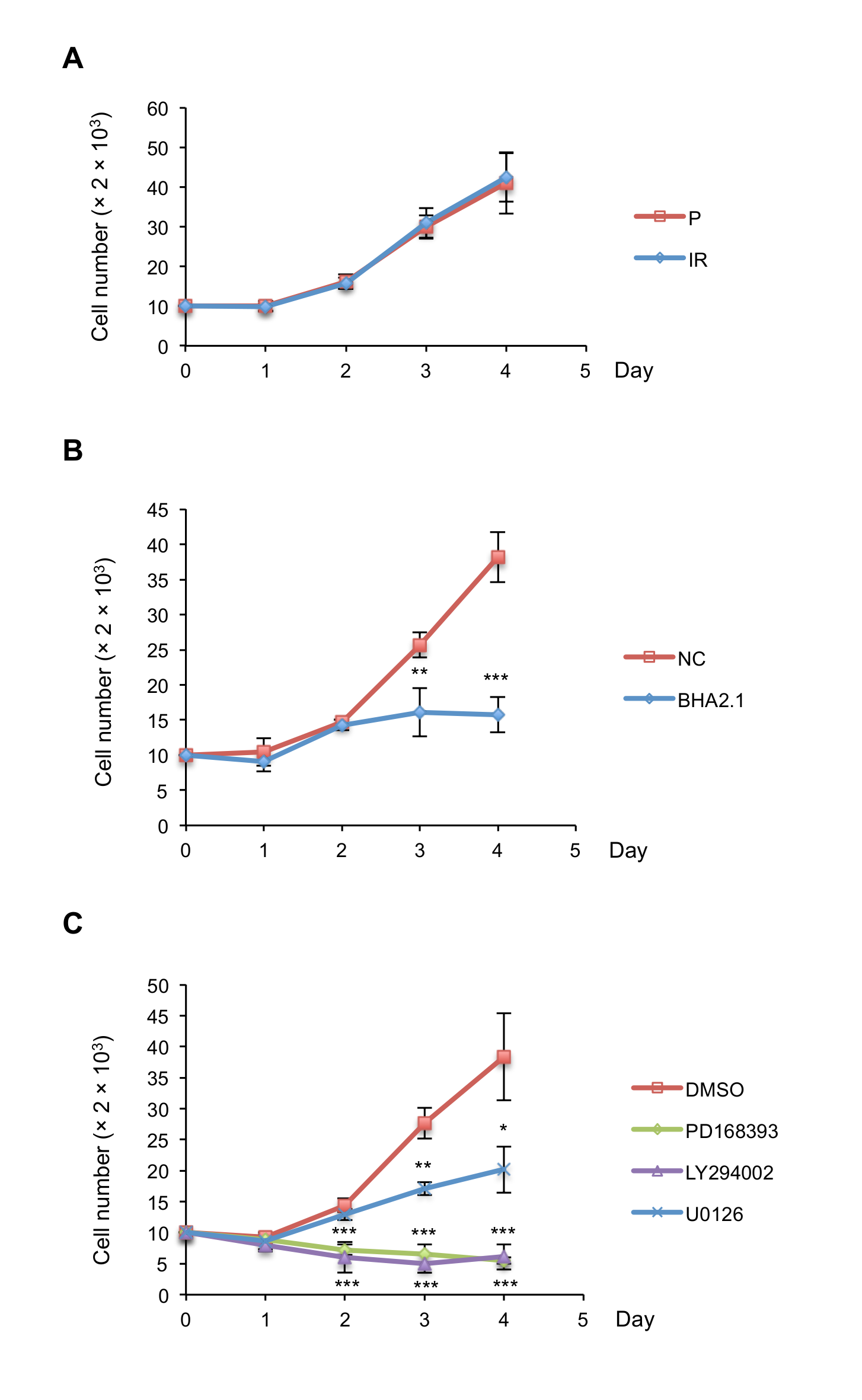

Supplement: Figure S1 — Proliferation analysis. (A) 2×10^4^ P and IR cells were cultured in 3D collagen gel in 24-well plate for 1 to 4 days to evaluate their proliferation rates. (B) 2×10^4^ IR cells were cultured in 3D collagen gel in 24-well plate for 1 to 4 days, treated with non-function blocking antibody for integrin α2β1 (NC), or treated with function blocking antibody for integrin α2β1 (BHA2.1). (C) 2×10^4^ IR cells were cultured in 3D collagen gel in 24-well plate for 1 to 4 days, treated with PD168393, LY294002 or U0126 versus DMSO control. Cell numbers are presented as mean values ± S.D (*p<0.05, **p<0.01, ***p<0.001) from 3 independent experiments performed in triplicate. (TIF) [file pone.0070905.s001.tif]
